# Fish consumption and brain structure: a comprehensive systematic review of observational studies

**DOI:** 10.1007/s40520-026-03363-x

**Published:** 2026-04-11

**Authors:** Justyna Godos, Giuseppe Caruso, Agnieszka Micek, Alberto Dolci, Zoltan Ungvari, Andrea Lehoczki, Lisandra Leon, Evelyn Frias-Toral, Andrea Di Mauro, Mario Siervo, Michelino Di Rosa, Giuseppe Grosso

**Affiliations:** 1 Department of Biomedical and Biotechnological Sciences, University of Catania, Catania, 95123 Italy; 2 Departmental Faculty of Medicine, UniCamillus-Saint Camillus International University of Health and Medical Sciences, Rome, Italy; 3 IRCCS San Camillo Hospital, Venice, Italy; 4 Statistical Laboratory, Faculty of Health Sciences, Jagiellonian University Medical College, Cracow, 31-501 Poland; 5 Sustainable Development Department, Bolton Food SpA, Milan, 20124 Italy; 6 Vascular Cognitive Impairment, Neurodegeneration and Healthy Brain Aging Program, Department of Neurosurgery, University of Oklahoma Health Sciences Center, Oklahoma City, OK USA; 7 Stephenson Cancer Center, University of Oklahoma, Oklahoma City, OK USA; 8 Oklahoma Center for Geroscience and Healthy Brain Aging, University of Oklahoma Health Sciences Center, Oklahoma City, OK USA; 9 Department of Health Promotion Sciences, College of Public Health, University of Oklahoma Health Sciences Center, Oklahoma City, OK USA; 10 International Training Program in Geroscience, Doctoral College/Institute of Preventive Medicine and Public Health, Semmelweis University, Budapest, Hungary; 11 Universidad Internacional Iberoamericana, Campeche, 24560 México; 12 Universidade Internacional do Cuanza, Cuito, Bié, Angola; 13 Fundación Universitaria Internacional de Colombia, Bogotá, Colombia; 14 School of Medicine, Universidad Espíritu Santo, Samborondón, 0901952 Ecuador; 15 Division of Research, Texas State University, 601 University Dr, San Marcos, TX 78666 USA; 16 Curtin-Chulalongkorn Collaborative Centre for Nutrition and Food Research and Education, Curtin University, Perth, WA Australia; 17 Faculty of Health Sciences, School of Population Health, Curtin University, Perth, WA 6102 Australia; 18 Curtin Dementia Centre of Excellence, Enable Institute, Curtin University, Perth, WA 6102 Australia; 19 Curtin Medical Research Institute (CMRI), Curtin University, Perth, WA 6102 Australia

**Keywords:** Fish, Brain structure, White matter volume, MRI

## Abstract

**Background:**

Age-related structural changes in the human brain, including cortical atrophy, reductions in grey and white matter volumes, and the accumulation of small vessel–related lesions such as white matter hyperintensities (WMH) and cerebral microbleeds, represent critical biological substrates underlying cognitive decline and dementia. Fish consumption has been associated with slower cognitive decline and reduced risk of dementia, but a comprehensive evaluation of its relation with brain structures is lacking.

**Aims:**

The aim of this study was to systematically review current scientific literature providing evidence of relation between fish intake and brain structures in human studies.

**Methods:**

Studies indexed in two major electronic databases have been screened based on a combination of keywords and MeSH terms. Studies were eligible whether they assessed fish consumption in relation to brain structures in the adult populations.

**Results:**

A total of 24 studies conducted predominantly on older adults met inclusion criteria. Most brain volume measures were obtained via magnetic resonance imaging (MRI) procedures. Higher fish consumption was associated with reduced severity of white matter hyperintensities (a biomarker of cerebral small vessel disease and white matter damage) and cerebral micro-bleed, preservation of certain brain areas volumes (i.e., hippocampus, temporal lobe and periventricle white matter) and cortical thickness of specific areas (i.e., precuneus, parietal, and cingulate grey matter), among others, compared to lower intake. Some analyses found no association and isolated findings suggested possible adverse associations that were not consistently replicated. Studies reporting null findings may underline the possible relevance of the overall diet (i.e., adherence to the Mediterranean diet).

**Conclusions:**

Inclusion of fish in a healthy and balanced diet is associated with better white matter grades on MRI and slower progression of white matter hyperintensities and reduction of vascular-related lesions of the aging brain, suggesting a potential role in preventing neurocognitive deterioration. Heterogeneity across studies underscores the need for additional studies.
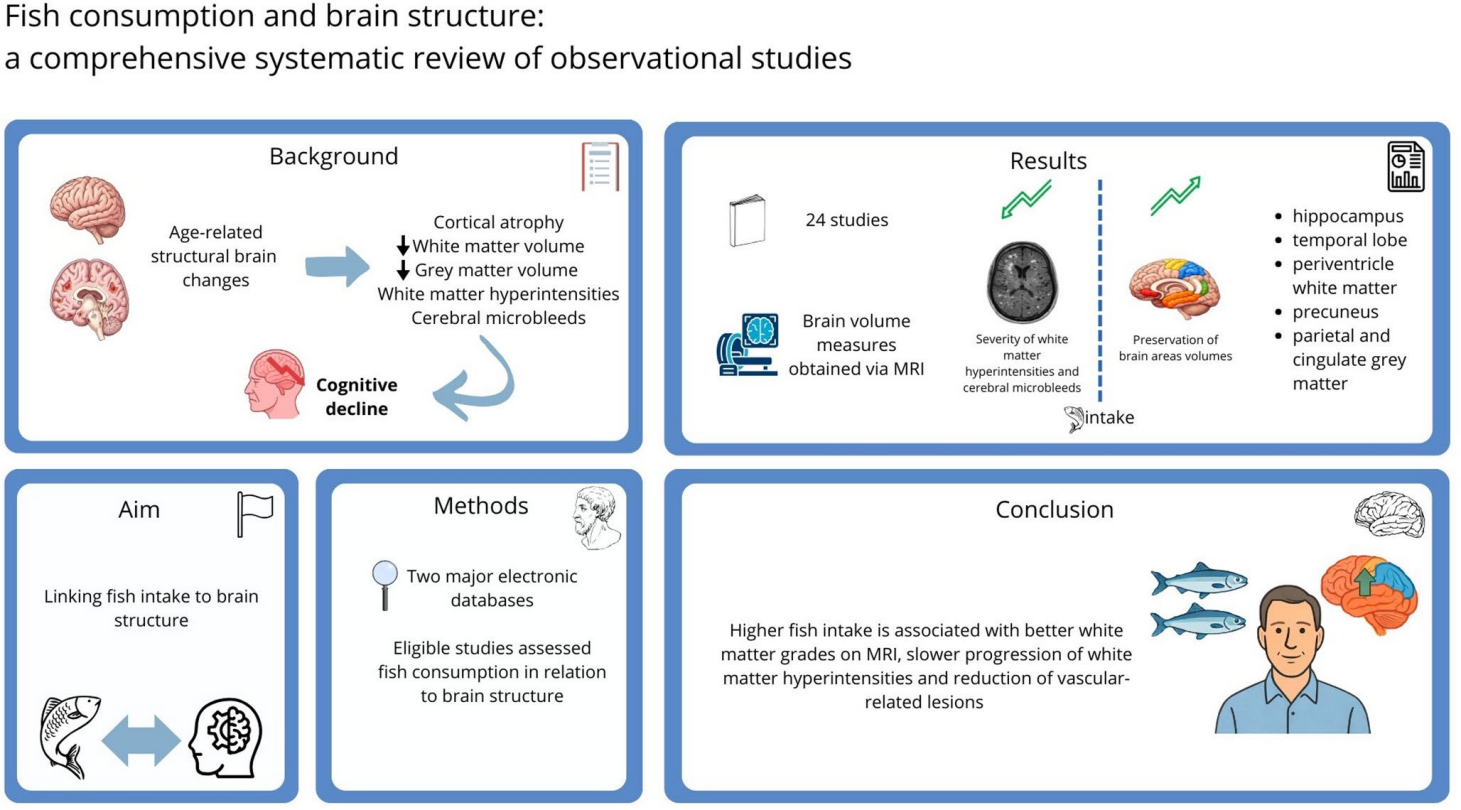

**Supplementary Information:**

The online version contains supplementary material available at 10.1007/s40520-026-03363-x.

## Introduction

Population aging has become a defining demographic trend of the 21st century, with the proportion of individuals aged 65 years and older increasing at an unprecedented pace worldwide [1]. As longevity rises, the global burden of age-related neurological disorders is escalating, particularly dementia and milder forms of cognitive impairment [2]. Current epidemiological estimates indicate that the number of people living with dementia is projected to more than triple by 2050, driven predominantly by growth in older age groups and increased survival with chronic diseases [3]. This surge carries profound public health implications, as cognitive decline not only compromises quality of life and independence but also poses substantial societal and economic costs [4]. Importantly, structural brain changes such as cortical thinning, hippocampal atrophy, white matter deterioration, and microvascular lesions emerge decades before clinical symptoms [5, 6], emphasizing the need for early preventive strategies that target modifiable risk factors. In this context, diet and specifically fish consumption as part of a nutrient-dense dietary pattern, has gained attention as a potentially accessible avenue for mitigating the neurobiological processes that underlie cognitive aging [7].

Age-related structural changes in the human brain, including cortical atrophy, reductions in grey and white matter volumes, and the accumulation of small vessel–related lesions such as white matter hyperintensities (WMH) and cerebral microbleeds, represent critical biological substrates underlying cognitive decline and dementia [8]. Nutritional factors have emerged as potentially modifiable determinants of these neuroanatomical trajectories, with particular interest on dietary patterns characterized by frequent fish and seafood consumption [9]. Comprehensively, fish consumption has been associated with various health benefits, including (but not limited to) better cardiovascular health and reduced risk of certain cancers [10, 11]. Regular intake of fish has been repeatedly associated with slower cognitive decline and reduced dementia risk in epidemiological studies [12], a relationship thought to be mediated by the unique nutrient profile of fish, most notably long-chain omega-3 polyunsaturated fatty acids (PUFAs), such as docosahexaenoic acid (DHA) and eicosapentaenoic acid (EPA), high-quality protein, B-vitamins, vitamin D, selenium, and other bioactive compounds [13]. Notably, the nutrient content together with the overall food matrix of fish may explain to a better extent its potential role on brain health, possibly through improvement in brain structures [14, 15].

Given this background, exploring current evidence reporting a link between fish consumption and brain morphology is essential for interpreting the observed imaging findings and for clarifying whether fish intake contributes directly to neuroprotection or acts as a proxy for broader dietary patterns. The following section expands on potential biological mechanisms through which fish intake may influence brain volume preservation, microvascular integrity, and resistance to age-related structural deterioration.

## Methods

The design and reporting of this study followed the Meta-analyses Of Observational Studies in Epidemiology (MOOSE) guidelines (Supplementary Table 1) [16]. The systematic review protocol was registered in the PROSPERO International Prospective Register of Systematic Reviews database (ID: CRD42024501232, at https://www.crd.york.ac.uk/prospero/*).*

### Search strategy and study selection

To identify potentially eligible studies, a systematic literature search of PubMed and Scopus databases was performed from their inception up to September 2025. The search strategy was based on the combination of the relevant keywords inserted as text words and MeSH terms, related to fish, seafood and shellfish and structural brain parameters (Supplementary Table 2). Eligibility criteria for the systematic review and meta-analysis were specified using the PICOS criteria (Supplementary Table 3). Studies were eligible if they met the following inclusion criteria: (1) reporting on adult population; (2) had observational design (cohort studies, cross-sectional studies, case-control studies); (3) reported exposures to habitual fish, seafood, or shellfish consumption assessed through either 24-h recalls, food frequency questionnaires (FFQs), or dietary diaries; (4) investigated structural brain parameters as outcome; (5) reported statistical data on the measure of the relation between exposure and outcome. Although no language restriction criteria were applied, only studies in English were considered for inclusion in this systematic review. Reference lists of all eligible studies and relevant literature were also examined for any additional studies not previously identified. The systematic literature search and study selection were performed by two independent investigators (J.G. and A.M.) and any incongruity was resolved through a discussion and reaching consensus.

### Data extraction and quality assessment

Data from all included studies were extracted using a standardized electronic form including the following information : first author name, publication year, study design and location, population age and sex, sample size, details on the assessment method of dietary habits, details on the exposure, details on the assessment method of the outcome of interest, outcome of interest, main findings of the study.

The quality of each included study was rated according to the Newcastle-Ottawa Quality Assessment Scale, consisting of 3 domains of quality (selection, comparability, and outcome) and assessing specific study characteristics depending on the type of study design [17]. In detail, studies scoring over 5 and 7 points for cross-sectional and prospective studies, respectively, were identified as being of good/high quality. Two investigators (J.G. and A.M.) extracted the data and assessed the methodological quality independently and any incongruity was resolved through a discussion and reaching consensus.

## Results

### Study selection and main characteristics

The systematic search identified 1806 potentially relevant articles (Supplementary Fig. 1). After removing 526 duplicates, 1280 records were screened based on titles and abstracts. Of these, 1237 articles were excluded, leaving 43 articles for full-text assessment. Following full-text evaluation, 20 studies met the eligibility criteria. An additional four studies were identified through hand-searching of full texts and relevant literature, resulting in a final inclusion of 24 studies (14 retrospective/cross-sectional and 10 prospective studies). The overall study quality of cross-sectional studies was rather acceptable while studies with a prospective design had a substantial high quality.

Across the studies included in the systematic review (Table 1), research designs were predominantly prospective cohort studies, with several cross-sectional analyses and fewer retrospective investigations. Study populations were largely composed of community-dwelling older adults, typically aged 60 years or above, although a small number of cohorts included middle-aged adults or broader adult age ranges. Most studies were embedded within well-established population cohorts, such as the Cardiovascular Health Study (CHS), Northern Manhattan Study, Rotterdam Study, UK Biobank, Women’s Health Initiative Memory Study, and various European longitudinal aging cohorts. Sample sizes varied widely, from fewer than 100 participants to over 30,000 individuals, reflecting substantial methodological heterogeneity. Dietary assessment methods primarily relied on FFQs or repeated 24-hour dietary recalls, with some studies additionally quantifying biomarkers of omega-3 status. Brain structure outcomes were consistently evaluated using magnetic resonance imaging (MRI), with measures including global and regional brain volumes, cortical thickness (CT), WMH, microvascular lesions (including microbleeds and subclinical infarctions), ventricular size, hippocampal volumes, and diffusion-derived indices of white matter integrity. Together, these studies provide a broad, multi-cohort depiction of how fish intake may relate to structural markers of brain aging.

### Summary of retrospective/cross-sectional studies

Only one retrospective cohort study conducted within the Project Y cohort in the Netherlands included individuals with multiple sclerosis (MS) of the same birth year (1966), both males and females (*n* = 361; mean age 53.1 years) and healthy controls, also including males and females (*n* = 125; mean age 52.9 years). Dietary intake was assessed using individual dietary questions, with a focus on oily fish consumption. Brain outcomes were evaluated through MRI, assessing including normalized mean upper cervical cord area (MUCCA), normalized total brain volume (NBV), normalized white matter volume (NCGMV), normalized deep grey matter volume (NDGMV), normalized white matter volume (NWMV), normalized thalamic volume (NThalV), normalized cerebellar grey matter volume (NCbV). Results indicated that no significant associations were observed between oily fish intake and MRI-derived brain volumes in either MS patients or healthy controls [18].

Several other cross-sectional investigations also assessed associations between fish intake and brain structure. In a cross-sectional study, high-resolution structural MRI images were collected from 674 elderly adults (mean age 80.1 years) without dementia, who were participants in a community-based multiethnic cohort. Dietary information was obtained through a FFQ. Total brain volume (TBV), total grey matter volume (TGMV), total white matter volume (TWMV), mean cortical thickness (mCT), and regional volumes or thickness measures were derived from the MRI scans using the FreeSurfer software. Consuming fish at least 3 to 5 ounces (about 85–142 g) per week was linked to higher mean mCT (β = 0.019, *p* = 0.03) and greater TGMV (β = 7.06, *p* = 0.006), after accounting for age, sex, education, ethnicity, BMI, diabetes, and overall cognitive performance [19]. Another cross-sectional study included 672 cognitively normal participants aged 70 years and older from the Mayo Clinic Study of Aging (MCSA). Dietary intake was assessed using a modified Block 1995 Revision of the Health Habits FFQ (128 items). Brain structural outcomes were evaluated by MRI to estimate average CT across parietal, temporal, frontal, and occipital lobes. Results showed that higher fish intake was associated with greater CT in the precuneus, superior parietal, posterior cingulate, supramarginal, middle temporal, fusiform, and inferior parietal regions, as well as with average lobar CT, and was marginally associated with Alzheimer’s disease signature, temporal, and frontal CT [20]. A larger study embedded in the Rotterdam Study included 4,213 participants (mean age 65.7 y) from a community-dwelling cohort in the Netherlands aged > 45 years free of dementia. Dietary intake was assessed using a validated, self-administered, semiquantitative 389-item FFQ. Brain MRI was performed, applying automated brain tissue classification to quantify total brain, grey matter, white matter, hippocampal, and white matter lesion volumes. Results showed that higher adherence to the Dutch dietary guidelines, particularly consuming fish ≥ 100 g/week, was associated with larger total and white matter volumes and with a marginally higher hippocampal volume [21]. Similarly, a cross-sectional study included 76 healthy participants (45 females, 31 males) aged 31–59 years, recruited in Kyoto. Fish consumption was assessed using the brief self-administered diet history questionnaire, while brain structure was examined using neuroimaging-derived measures: the grey-matter brain healthcare quotient (GM-BHQ) and the fractional anisotropy brain healthcare quotient (FA-BHQ). Results showed that GM-BHQ scores were not correlated with fish intake (*r* = -0.014, *p* > 0.05), whereas higher fish intake was significantly associated with higher FA-BHQ scores (β = 0.309, *p* = 0.016). ANCOVA further indicated that adjusted mean FA-BHQ scores increased with greater fish intake, differing significantly between “<1 time/week” and “2 times/week” (97.6 vs. 94.4, *p* = 0.005) and between “<1 time/week” and “>2 times/week” (97.2 vs. 94.4, *p* = 0.034) [22]. In contrast, null findings were reported in a cross-sectional analysis from the German multicenter DELCODE cohort including 512 individuals (mean ± SD age = 69.49 ± 5.86 years; 270 females). Fish consumption was assessed using the European Prospective Investigation into Cancer and Nutrition (EPIC) FFQ, and voxel-based morphometry was applied to examine the relationship between grey matter volume (GMV) and fish intake. The results showed no significant association between fish intake and medial temporal lobe (MTL) volume [23].

Some studies also investigated the role of fish consumption in cerebral microbleeds (lesions that reflect cerebral small vessel disease and age-related tissue damage) and other alterations of the normal physiology of vascular brain structure. An early study conducted on 286 deceased participants of the Memory and Aging Project clinical neuropathological cohort study, 2004–2013 (mean age of death 89.9 years) previously assessed for dietary intakes via a semiquantitative FFQ reported that neither higher fish intake (> 1 meal/wk) nor total EPA + DHA dietary intake were associated with microinfarcts, although the association with AD neuropathology markers was significant in APOE e4-positive [24]. In contrast, a study conducted on 293 community-dwellers aged > 60 years enrolled in the Atahualpa Project reporting a relatively high fish consumption (mean 8.8 servings/wk) showed an inverse relationship between cerebral microbleeds and fish consumption (*p* < 0.001), with higher predictive margins for individuals in the lowest (< 4.3) than for those in the highest (> 13.1) quintile of fish servings (17.4 vs., 2.3%, *p* < 0.001) [25]. From the same extended cohort (*n* = 590, mean age 71.1 years), the authors also published a report on the association between oily fish intake and the presence of moderate-to-severe WMH (OR: 0.89; 95% C.I.: 0.85–0.94; *p* < 0.001), with predictive margins revealing an almost linear inverse relationship between quartiles of oily fish intake and probabilities of WMH severity [26].

### Summary of prospective studies

Among prospective studies, a cohort study conducted within the Three-City (3 C) Dijon Study, a population-based study in France, included 1,623 men and women aged ≥ 65 years (mean age 72.3 years) who were free from dementia, stroke, or any history of hospitalized cardiovascular disease at baseline. Dietary intake was evaluated using a FFQ. Brain outcomes were assessed through MRI, focusing on the shared dimension across three MRI phenotypes (WMH load, covert brain infarcts, and dilated perivascular spaces), computed as the first component from a factor analysis of mixed data (FAMD) of these markers. Results showed that a higher frequency of fish consumption was significantly associated with a lower global cerebrovascular disease (CVD) burden (p for trend < 0.001). Compared with participants consuming fish less than once per week, those eating fish 2–3 times per week and ≥ 4 times per week had a 0.19-unit (β = -0.19, 95% CI: -0.37 to -0.01) and 0.30-unit (β = -0.30, 95% CI: -0.57 to -0.03) lower global CVD burden, respectively [27]. Similar results concerning WMH were retrieved from a prospective analysis of the Atahualpa Project Cohort, in which an inverse relationship between oily fish intake and WMH progression (IRR: 0.89; 95% CI: 0.84–0.95; *p* < 0.001) was reported [28]. A prospective cohort study using data from the Women’s Health Initiative Memory Study–Magnetic Resonance Imaging (WHIMS-MRI), including 1,371 dementia-free elderly women, investigated whether red blood cell long-chain omega-3 polyunsaturated fatty acid levels modify the association between fine particulate matter (PM2.5) exposure and brain structure. Omega-3 PUFA levels and non-fried fish consumption was assessed at baseline using a semiquantitative FFQ. Results showed that higher non-fried fish consumption was associated with greater white matter volume, particularly in the temporal lobe [29]. A multicentric, longitudinal, observational study conducted in Germany within the Deutsches Zentrum für Neurodegenerative Erkrankungen (DZNE)-Longitudinal Cognitive Impairment and Dementia (DELCODE) study included 938 men and women, comprising healthy older adults and individuals at risk for AD (mean age 70.8 years). Dietary intake was assessed using the validated EPIC-FFQ, with specific evaluation of fish consumption. Brain outcomes were examined through MRI, focusing on left and right hippocampus volumes. Results showed that greater fish consumption was significantly associated with larger bilateral hippocampus volumes (standardized regression coefficients: β = 0.09, *p* < 0.001 for the left hippocampus and β = 0.09, *p* = 0.01 for the right hippocampus) [30]. Another report from the CHS, included 2,863 adults aged ≥ 65 years. Dietary intake was assessed using the National Cancer Institute FFQ, evaluating consumption of tuna, other broiled or baked fish, and fried fish. Intakes of EPA and DHA were estimated and adjusted for total energy. Plasma phospholipid EPA + DHA concentrations correlated strongly with tuna and other baked/broiled fish intake (*r* = 0.51), but not with fried fish (*r* = 0.04). MRI was used to assess brain structure, with neuroradiologists blinded to participants’ clinical data. After adjusting for potential confounders, mean white-matter grades (WMGs) decreased linearly with higher tuna/other-fish intake from 2.50 (< 1 serving/month) to 2.20 (≥ 3 servings/week), corresponding to a 10.6% lower mean grade. Each higher consumption category was associated with a 3.8% reduction in mean WMG. No association was found for fried fish intake [31]. In contrast, a report from the same cohort focused on 260 cognitively normal older adults, showed that regular weekly consumption of baked or broiled fish was associated with greater GMV in several regions involved in memory and cognitive processing, including the hippocampus, precuneus, posterior cingulate, and orbitofrontal cortex. In particular, eating fish at least once per week corresponded to an approximate 4.3% larger volume in the orbitofrontal and anterior cingulate areas, and about a 14% increase in the MTL volume [32]. Concerning microbleeds, a study conducted on prospective data retrieved from the CHS (*n* = 2313 individuals aged > 65 years) undergoing voluntary MRI scans for subclinical infarctions showed an inverse association between fish consumption assessed via FFQ and outcome of interest, although the relation was attenuated after adjustment for DHA [33].

Some studies reported contrasting and null results. A longitudinal prospective study conducted within the Cognitive Reserve (CR) and Reference Ability Neural Network (RANN) cohorts included 183 cognitively intact adults (men and women) aged 20–80 years (mean age 53.2 years). Dietary intake was assessed using a semiquantitative FFQ, with a specific focus on fish consumption. Brain outcomes were evaluated using MRI to assess WMH. Results indicated that higher fish consumption appeared to be associated with a greater increase in WMH volume; however, this association did not reach statistical significance (β = 0.071, 95% CI: -0.002 to 0.143, *p* = 0.056) [34]. A prospective cohort study assessing the association between adherence to a Mediterranean-type diet and changes in brain MRI volumetric measures and mean CT among 562 participants from the Lothian Birth Cohort of 1936. Fish intake was estimated using the Scottish Collaborative Group 168-item FFQ. Brain structural outcomes were assessed through MRI. Results showed that fish consumption was not significantly associated with TBV or GMV at age 73, nor with changes in these measures between ages 73 and 76 [35]. Another prospective cohort study, the Northern Manhattan Study, was designed to determine stroke incidence, risk factors, and prognosis in a multi-ethnic urban population. The study included 966 clinically stroke-free adults who underwent brain imaging on a 1.5T MRI system at the Hatch Research Center. At baseline, participants completed a modified Block National Cancer Institute FFQ to assess dietary intake, including fish consumption as part of the Mediterranean diet. After simultaneous adjustment for other Mediterranean diet components, age at MRI, sex, ethnicity, education, and physical activity, no significant difference in log-transformed WMH volume was observed between individuals with fish consumption above versus below the sex-specific median (β = 0.022, SE = 0.69). These findings suggested that adherence to the fish component of the Mediterranean diet was not associated with WMH burden [36]. Similarly, another prospective cohort study included 194 cognitively healthy individuals participating in the Prospective Investigation of the Vasculature in Uppsala Seniors (PIVUS) cohort. Dietary intake was assessed using a 7-day dietary registration, and at the age of 75, participants completed the Swedish translation of the Seven Minute Screening (7MS) test, a clinical tool used to evaluate dementia and cognitive decline. The test comprised four brief cognitive assessments, including the Benton temporal orientation task. High-resolution 3D T1-weighted “Turbo Field Echo” (TFE) MRI scans were acquired to assess brain structure. No association was found between fish consumption and changes in brain parameters [37]. Contrasting results have also been reported in the Lothian Birth Cohort Study 1936, in which 882 individuals (mean age 72 years, SD ± 0.8) were followed up for 3 years, part of them assessed for cognitive status (*n* = 866) and part (*n* = 700) underwent brain MRI scans to measure ventricular, hippocampal, and normal and abnormal tissue volumes. Within this subgroup, 189 participants were categorized into four distinct groups representing extreme or intermediate patterns of iodine intake, with no overlap between groups. The study examined differences in brain structure and cognitive performance between those with low or low intake of iodine-rich foods and those with high consumption, particularly dairy and fish. Oily fish intake was significantly associated with enlargement of the left lateral ventricle (standardized β = 0.20, *p* = 0.03), although this association became non-significant when outliers were excluded. Intake of fish products was also significantly associated with WMH (standardized β = 0.28, *p* = 0.002) [38]. An earlier study conducted within the UK Biobank included 10,938 men and women aged 45–80 years. Dietary intake was assessed using up to five 24-hour dietary recalls per year, focusing on shellfish consumption as the primary exposure variable. Brain outcomes were evaluated through MRI, assessing TBV and WMH. Results showed that higher shellfish intake was associated with greater white matter lesion load [39]. Also another analysis including 30,375 participants, after excluding individuals with extreme WMH to reduce bias and heterogeneity in brain age prediction. The study employed both an exposome-wide association study (XWAS) and a genome-wide association study (GWAS), highlighting the multifactorial nature of brain aging. WM BAG was predicted using machine learning based on fractional anisotropy measurements. In the XWAS, 107 environmental and lifestyle variables were analyzed, grouped into five categories: electronic device use, smoking, diet, alcohol, and sun exposure. Oily fish intake was associated with accelerated brain aging after adjustment for age, sex, and BMI (β = 0.08, BH-adjasted *p* < 0.001). A multivariate analysis including a broad range of covariates confirmed this finding (β = 0.04, BH-adjasted *p* = 0.02) [40]. Conversely, a newer report from the same cohort including a subgroup of 2,723 participants aged 40–69 years (mean age 52.7 years, men and women). Dietary intake was assessed via 24-hour dietary recalls, with specific evaluation of seafood consumption, including oily fish, white fish, tinned tuna, and breaded or battered fish. Brain outcomes were evaluated through MRI, assessing total hippocampus volume (THV), right hippocampus volume (RHV), and left hippocampus volume (LHV). Results showed that individuals with a higher intake of seafood exhibited greater longitudinal increases in THV and RHV, but not in LHV, compared with those reporting no seafood consumption (THV, β: 0.004514, *p* < 0.05; RHV, β: 0.005527, *p* < 0.05; LHV, β: 0.003269, *p* = 0.2929) [41].

## Discussion

The present study reported accumulating neuroimaging evidence indicating that fish intake may exert measurable effects on brain structure. Studies in community-dwelling older adults and large population-based cohorts using MRI have associated higher fish intake with preserved hippocampal and temporal lobe volumes, greater CT in parietal and cingulate regions, improved white matter integrity, and lower burdens of WMH and microbleeds. Across the studies included in this review, the most consistent pattern of findings relates to the association between fish consumption and markers of white matter integrity and cerebrovascular health. Although a few reported divergent or adverse patterns, the clearest and most reproducible benefits were observed for outcomes reflecting small-vessel disease burden, white matter microstructure, and lesion accumulation, rather than broad measures of global or TBV. This coherence across multiple studies suggests that fish intake may exert its strongest neuroprotective effects through vascular and myelin-related pathways. Compared with white matter findings, associations involving global brain volume, total grey matter, or ventricular measures were notably weaker and more inconsistent. Many studies reported null associations in these domains, suggesting that fish consumption may not exert broad neuroprotective effects detectable at the whole-brain level. Instead, the pattern pointing toward a more targeted influence on vasculature-dependent and myelin-rich structures aligns with well-established biological pathways through which omega-3 fatty acids enhance endothelial health, reduce inflammation, support myelin integrity, and mitigate ischemic and hypoxic injury processes, all mechanisms with particularly strong relevance to white matter and small-vessel function. However, other nutritive components of fish might provide further rationale for its potential benefits toward brain health. Nonetheless, the available findings are heterogeneous, and some investigations report null or context-dependent associations, underscoring the need for a deeper understanding of the biological pathways that may explain how fish consumption could influence neurostructural outcomes.

The mechanistic pathways through which fish consumption may influence brain structure are multifaceted and likely operate through interrelated biological processes. Fish is a major dietary source of long-chain omega-3 PUFAs, such as DHA and EPA, which play essential roles in neuronal membrane composition, synaptic function, and neuroinflammation [42]. One of the most prominent mechanistic explanations involves maintenance of white matter integrity and protection of small cerebral vessels, which aligns closely with the most consistent findings across studies: DHA is an abundant fatty acid in myelin and oligodendrocyte membranes, contributing to membrane fluidity and electrical conductivity, with experimental studies indicating that DHA enhances oligodendrocyte differentiation and reduces demyelination under oxidative or inflammatory stress [43, 44]. EPA and DHA also exert anti-inflammatory and antithrombotic effects, partly through modulation of eicosanoid synthesis, inhibition of pro-inflammatory cytokines, and incorporation into endothelial cell membranes [45]. These mechanisms may reduce the development or progression of WMH, microvascular lesions, and blood–brain barrier dysfunction, conditions strongly implicated in age-related cognitive decline [46, 47]. The observed associations between fish intake and lower cerebrovascular disease burden, better fractional anisotropy, and preserved temporal lobe white matter in several cohorts align well with these mechanistic pathways [22]. A second pathway relates to region-specific grey matter preservation, particularly within the MTL, cingulate cortex, and parietal regions. DHA is an essential structural component of neuronal membranes in cortical and hippocampal neurons and is involved in synaptic vesicle formation, neurotransmitter release, and dendritic spine maintenance [48, 49]. Higher DHA availability supports synaptogenesis and long-term potentiation, processes fundamental for memory and executive function [50, 51]. Fish also contains micronutrients important for neurodevelopment and maintenance, including vitamin D, selenium, iodine, and high-quality protein, each of which contributes to neurotrophic signaling, oxidative defense, thyroid hormone synthesis, and neuronal repair [52]. In combination, these nutrients may preferentially support regions with high metabolic demands and susceptibility to early neurodegeneration, thereby explaining why several studies observed larger hippocampal or posterior cortical volumes among individuals with higher fish intake [19, 21]. Cerebrovascular protection is an additional mechanism linking fish consumption to structural brain outcomes. Omega-3 PUFAs have been shown to improve endothelial function, lower triglycerides, reduce platelet aggregation, and stabilize atherosclerotic plaques [53–55]. Improved vascular health may enhance cerebral blood flow, reduce chronic hypoperfusion, and mitigate lacunar infarction and perivascular space dilation, mechanisms that directly contribute to WMH and small-vessel disease markers captured in MRI studies [27]. The stronger associations observed in populations with higher vascular risk burden lend further support to the hypothesis that fish consumption may be particularly protective in the context of age-related or lifestyle-related vascular stress. Another mechanistic pathway involves attenuation of oxidative stress and neuroinflammation, both of which contribute to neuronal atrophy and white matter deterioration. Selenium, a trace mineral abundant in fish, is a cofactor for glutathione peroxidases, key enzymes in antioxidant defense [56]. Moreover, EPA and DHA are precursors to resolvins and protectins, specialized pro-resolving lipid mediators that reduce microglial activation and promote resolution of inflammation [57, 58]. These anti-inflammatory effects may help preserve neuronal and glial cell function over time, contributing to structural stability [59]. Beyond nutrients, fish consumption may also serve as a marker of broader dietary and lifestyle patterns that support brain health. In fact, fish represents a cornerstone for healthy dietary patterns, such as the Mediterranean diet, which is characterized by an overall high consumption of vegetables, legumes, nuts, and whole grains, and moderate intake of meat-derived and processed foods [60]. Such a dietary pattern has been extensively associated with lower risk of cognitive decline and dementia, possibly due to neuroprotective effects through reduced inflammation, better vascular health, and lower exposure to neurotoxic dietary components [61]. Therefore, part of the observed associations between fish and brain structure may be secondary to overall diet quality, underscoring the need for studies disentangling fish-specific effects from dietary synergies.

An important limitation in the current literature is the substantial heterogeneity across studies investigating the association between fish consumption and structural brain outcomes Table 1. This heterogeneity spans multiple domains (i.e., study design, participant characteristics, dietary assessment methods, MRI outcomes, and analytical approaches) which collectively complicate the derivation of uniform conclusions. Across cohorts, findings ranged from robust protective associations involving white matter integrity and region-specific GMVs to complete absence of associations. Such variability underscores the complexity of isolating the influence of a single dietary component within diverse populations and methodological frameworks. Differences in study design constitute a major source of heterogeneity. Prospective cohorts, such as those derived from the CHS, the Three-City Dijon Study, and the UK Biobank, generally provided more robust temporal inferences than cross-sectional studies. Yet even among prospective studies, results diverged markedly: some demonstrated significant neuroprotective associations of fish intake with white matter lesion burden or hippocampal preservation, while others reported null or inconsistent findings despite similar follow-up durations. The inclusion of a retrospective cohort, such as the Project Y study in individuals with MS, added further variation, particularly given that disease-related neurodegeneration may overshadow dietary influences. Participant characteristics also varied widely, contributing additional heterogeneity. Many cohorts comprised older, community-dwelling adults, yet others included middle-aged individuals, mixed-age samples, or clinical populations. Differences in baseline cognitive status, vascular risk profiles, genetic susceptibility, socioeconomic background, and lifestyle factors may all modulate the observed associations between diet and neuroanatomical outcomes. For example, studies involving older adults with substantial vascular burden generally showed stronger associations between fish intake and white matter–related outcomes, whereas studies in healthier or younger populations more often reported null effects. Variation in dietary assessment methods further complicates comparability. Most cohorts relied on FFQs, which differ in item number, reference periods, and validation procedures. Some assessed intake repeatedly, whereas others relied on a single baseline measure. Importantly, studies differed in whether they considered total fish intake, oily fish intake, non-fried fish, shellfish consumption, or combined seafood categories, each of which may reflect distinct nutrient profiles and preparation methods. Finally, lack of adjustment for other dietary factors may also contribute to heterogeneity of results. Only a minority of studies incorporated objective biomarkers such as red blood cell omega-3 levels, which may more accurately reflect long-term intake, thus potentially explaining discrepancies with studies relying solely on self-report. Differences in MRI field strength, acquisition protocols, image processing pipelines, and analytic strategies introduce further sources of variability. Finally, the extent and handling of covariate adjustment varied broadly. Some studies included extensive adjustments for socioeconomic status, cardiovascular risk factors, and broader dietary patterns, whereas others included more limited sets of confounders. Such variability may lead to under- or over-adjustment, thereby contributing to divergent findings. In addition, the influence of lifestyle factors often correlated with fish consumption (such as physical activity, alcohol intake, or supplement use) may not have been uniformly accounted for. Altogether, the heterogeneous findings across these studies emphasize the need for careful interpretation and highlight the importance of methodological harmonization. The variability suggests that the association between fish intake and brain structure is unlikely to be uniform across all populations and outcomes but may depend on specific combinations of individual characteristics, dietary exposures, and neuroanatomical metrics. This heterogeneity also reinforces the need for standardized dietary assessment tools, harmonized imaging outcomes, and biomarker-based exposure measures to clarify the underlying relationships.

**Table 1:** The main characteristics of the included studies in the systematic-review reporting on the relation between fish consumption and structural brain parameters

Author, year, country	Cohort name, study design*	Population (sex, mean age)	Total population	Exposure	Exposure assessment method	Outcome	Outcome assessment method	Main findings
Virtanen [31], US	CHS (prospective cohort study)	Older adults (MF, ≥ 65 y)	2863	Tuna/other fish, fried fish intake	FFQ, SQ-FFQ	WMG	MRI	After adjustment for potential clinically relevant confounders, mean white-matter grades decreased linearly with increasing tuna/other-fish intake, from about 2.50 for < 1 serving/month to 2.20 for ≥ 3 servings/week. This corresponds to an approximately 10.6% lower mean grade for ≥ 3 servings/week compared with ≤ 1/month. On average, each higher consumption category was associated with about a 3.8% lower mean white-matter grade. Fried fish consumption was not associated with white matter grade.
Gardener [36], US	MRI sub-study of The Northern Manhattan Study (prospective cohort study)	Clinically stroke-free adults (MF, > 55 y)	966	Fish intake	Modified Block National Cancer Institute FFQ	WMH	MRI	After simultaneous adjustment for other components of the Mediterranean diet, age at MRI, sex, ethnicity, education, and physical activity, there was no significant difference in log-transformed WMH volume between individuals with fish consumption above versus below the sex-specific median, reflecting adherence versus non-adherence to that component of the Mediterranean diet (β = 0.022, SE = 0.69).
Titova [37], Sweden	Prospective Investigation of the Vasculature in Uppsala Seniors (prospective cohort study)	Cognitively healthy elderly individuals (MF, 70 y)	194	Fish intake	7-day dietary registration	TBV, GM, WM	MRI	No association was found between fish consumption and structural brain parameters.
Virtanen [33]	Cardiovascular Health Study, prospective (US)	Random adults from Medicare eligibility lists (MF, > 65 y)	2313	Fish intake	FFQ	Subclinical infarcts	MRI	The multivariable-adjusted ORs of prevalent subclinical infarct across categories of fish intake were 1, 0.95, 0.84, and 0.70 (p_for trend_=0.09). Adjustment for phospholipid DHA substantially attenuated the association; the ORs were 1, 0.98, 0.96, and 0.87 (p_for trend_=0.54).
Raji [32], US	CHS-CS (prospective cohort study)	Cognitively normal older adults (MF, 78.4y)	260	Broiled or baked fish intake	FFQ	WML	Brain MRI	Weekly consumption of baked or broiled fish was positively associated with grey matter volumes in several brain regions, including the hippocampus, precuneus, posterior cingulate, and orbitofrontal cortex. Specifically, consuming fish at least once per week was linked to a 4.3% increase in the size of orbitofrontal and anterior cingulate regions and a 14% increase in the volume of medial temporal lobe.
Gu [19], US	WHICAP (prospective cohort study - analysis cross-sectional)	Elderly Medicare beneficiaries (≥ 65 years) residing in northern Manhattan (MF, 80.1y)	674	Fish intake	SQ-FFQ	TBV, TGMV, TWMV, mean cortical thickness (mCT)	MRI	Fish intake ≥ 3–5 oz/week was associated with thicker mean cortical thickness (β = 0.019, *p* = 0.03) and with a greater TGMV (β = 7.06, *p* = 0.006), when adjusting for age, sex, education, ethnicity, BMI, diabetes, and mean cognition.
Del Brutto [25], Ecuador	Atahualpa Project Cohort (prospective cohort study - analysis)	Community-dwellers aged ≥ 60 years living in three neighboring rural villages of coastal Ecuador (MF 71.1y)	293	Fish intake	Specific questions on fish consumption	Cerebral microbleeds	MRI	An inverse relationship between cerebral microbleeds and fish consumption (*p* < 0.001) was found, with higher predictive margins for individuals in the lowest (< 4.3) than for those in the highest (> 13.1) quintile of fish servings (17.4 vs. 2.3%, *p* < 0.001).
Morris [24]	Memory and Aging Project (cross-sectional), US	Diseased Chicago residents of retirement communities and subsidized housing (MF, 89.9 y)	286	4 seafood items (tuna sandwich; fish sticks, cakes, or sandwich; fresh fish as a main dish; and shrimp, lobster, or crab)	Semiquantitative FFQ	Microbleeds	Brain autopsies and pathologic evaluations	Seafood consumption was not associated with microbleeds (for ≥ 1 meal/wk vs. < 1 meal/wk, OR = 0.98, 95% CI 0.49–1.96).
Del C Valdés Hernández [38], UK	Scottish Mental Survey (prospective cohort study)	Community-dwelling surviving members of the Scottish Mental Survey of 1947	189	White fish, oily fish, shellfish, fish products, canned fish, total fish intake	SCG-FFQ	Cerebellum WM R, Cerebellum Cortex R, Cerebellum WM L, Cerebellum Cortex L, Lateral Ventricle R, Lateral Ventricle L, 3rd Ventricle, 4th Ventricle, Subarachnoid space, Hippocampus R, Hippocampus L, WMH	MRI	Oily fish intake was significantly associated with lateral ventricle L enlargement (standardised β = 0.20, *p* = 0.03) but became non-significant when outliers were excluded; Fish products intake were significantly associated with WMH (standardised β = 0.28, *p* = 0.002)
Luciano [35], UK (Scotland)	The Lothian Birth Cohort of 1936 (prospective cohort study)	Community originating in the Edinburgh region of Scotland, comprising individuals born in 1936 (MF, 70y)	562	Fish intake	168-item FFQ	TBV, GMV	MRI	Fish consumption was not associated with significant differences in TBV or GMV at age 73, nor in the magnitude of their change between ages 73 and 76.
Staubo [20], US	MCSA (cross-sectional study)	Residents from Olmsted County, Minn., USA, aged 70–89y (MF, 79.8y)	672	Fish intake	128 items FFQ	CT of: Precuneus, Superior Parietal, Posterior Cingulate, Parietal, Spramarginal, Middle Temporal, Fusiform, Inferior Parietal, Average lobar, AD signature, Temporal, Frontal	MRI	Higher fish intake was associated with larger CT for precuneus (β = 0.018, *p* < 0.001), superior parietal (β = 0.014 p 0.01), posterior cingulate (β = 0.012, *p* = 0.03), parietal (β = 0.011, *p* = 0.01), supramarginal (β = 0.011, *p* = 0.04), middle temporal (β = 0.11, *p* = 0.04), fusiform (β = 0.010, *p* = 0.05), inferior parietal (β = 0.010, *p* = 0.04), average lobar (β = 0.009, *p* = 0.02) and average lobar CT, and was marginally associated with AD signature CT (*p* = 0.06), temporal (*p* = 0.06) and frontal (*p* = 0.08) CT
Croll [21], Netherlands	The Rotterdam Study (cross-sectional study)	Community-dwelling cohort in the Netherlands aged > 45 years (MF, 65.7y)	4213	Fish intake	389-item SQ-FFQ	Total Brain Volume, Grey Matter Volume, White Matter Volume, Hippocampus Volume	MRI	Higher adherence to Dutch dietary guidelines, with a fish consumption ≥ 100 g/wk, was associated with larger white matter volume (β = 4.04, 95% CI 1.20 to 6.87, *p* < 0.05) as well as a marginally significantly higher hippocampal volume (β = 0.05, 95% CI: -0.00 to 0.10)
Chen [29], US	WHIMS (prospective cohort study)	Free of dementia, community dwelling, postmenopausal hormone therapy women, 65-80y (F, 70y)	1371	Nonfried fish intake	SQ-FFQ	Total white matter, Association WMV, Frontal lobe WMV, Parietal lobe WMV, Temporal lobe WMV, Corpus callosum	MRI	Higher nonfried fish consumption was associated with a greater WMV in the temporal lobe (β ± SE = 0.59 ± 0.28, p 0.034 per interquartile increment = 0.14 servings/day)
Kokubun [22], Japan	Kyoto University cohort (Cross-sectional study)	Healthy participants,31–59 years aged (MF, 47.0y)	76	Intake of fish prepared as grilled, tempura, or fried (excluding sashimi, sushi, boiled fish, stews, soups, or miso soup)	BDHQ	GM-BHQ, FA-BHQ	MRI	GM-BHQ scores not correlated with fish intake (*r* = -0.014, *p* > 0.05); meanwhile regression analyses showed that fish intake was significantly associated with higher FA-BHQ scores (standardized regression coefficient β = 0.309, *p* = 0.016). ANCOVA indicated that adjusted mean FA-BHQ scores were higher for greater fish intake, differing significantly between “<1 time/week” and “2 times/week” (97.6 vs. 94.4, *p* = 0.005) and “<1 time/week” and “>2 times/week” (97.2 vs. 94.4, *p* = 0.034).
Ballarini [23], Germany	DELCODE (cross-sectional study)	Older adults (MF 69.49 y)	512	Fish intake	EPIC-FFQ	Mediotemporal lobe (MTL) volume (referring to the bilateral grey matter volume within the significant cluster identified in the ROI-based analysis)	MRI	No significant relationship was observed between fish intake and MTL volume.
Del Brutto [26], Ecuador	Atahualpa Project Cohort (prospective cohort)	Community-dwellers aged ≥ 60 years living in three neighboring rural villages of coastal Ecuador (MF 71.1y)	572	Oily fish (broiled or cooked) intake	Non-standardized questionnaire: self-reported habitual consumption, with portion sizes standardized by field personnel using local market weights	WMH	MRI	The amount of oily fish intake was inversely associated with the presence of moderate-to-severe WMH (OR = 0.89, 95% CI: 0.85–0.94 per oily fish intake serving, *p* < 0.001).
Klinedinst [39], UK	UK Biobank (prospective cohort study)	Adults (MF 45–80 y)	10,938	Shell-fish intake	24-h recall (up to 5 times/year)	TBV, WMH	MRI	Greater lesion load was seen with consuming more shell-fish.
Thomas [27], France	3 C Dijon study (Prospective cohort study)	Population-based cohort aged ≥ 65 years, without dementia, stroke, or history of hospitalized cardiovascular disease (MF, 72.3y)	1623	Fish intake	FFQ of 10 broad categories of foods recorded in 6 classes; fish intake was categorized in 4 classes	Composite outcome of WMH load, covert brain infarcts, and dilated perivascular spaces	MRI	A higher frequency of fish consumption was associated with a lower CVD burden (p for trend < 0.001). Compared with participants who consumed fish less than once per week, those eating fish 2–3 times per week and ≥ 4 times per week had a 0.19-unit (β = −0.19, 95% CI: −0.37 to − 0.01) and 0.30-unit (β = −0.30, 95% CI: −0.57 to − 0.03) lower global CVD burden, respectively
Del Brutto [28], Ecuador	Atahualpa Project Cohort (longitudinal prospective design)	Community-dwellers aged ≥ 60 years living in Atahualpa (MF 65.7y)	263	Oily fish (broiled or cooked) intake	Non-standardized questionnaire: self-reported habitual consumption at every annual door-to-door survey	WMH	MRI	After adjusting for demographics, education level, and vascular risk factors, the Poisson regression model revealed a significant inverse association between oily fish intake and WMH progression rate (IRR = 0.89, 95% CI: 0.84–0.95, *p* < 0.001 for each additional weekly serving). Participants in the highest tertile of fish intake had significantly lower WMH progression rates than those in the lower tertiles (IRR = 0.44; 95% CI: 0.26–0.77, *p* = 0.004)
Song [34], US	Cognitive Reserve (CR) study and Reference Ability Neural Network (RANN) study (longitudinal prospective design)	Cognitively intact adults aged 20–80 years (MF, 53.19 y)	183	Fish intake	SQ-FFQ	WMH	MRI	Higher fish consumption appeared to be associated with a greater increase in WMH, but the association did not reach statistical significance (β = 0.071, 95% CI: -0.002 to 0.143, *p* = 0.056)
Loonstra [18], Netherlands	Project Y Cohort (Retrospective cohort study)	People with MS of the same birth year 1966 (MF, 53.1y) and healthy controls (MF,52.9y)	486	Oily fish intake	Individual questions.	MUCCA, NBV, NCGMV, NDGMV, NWMV, NThalV, NCbV	MRI	No significant association was observed between oily fish intake and MRI-derived brain volumes in either MS patients or the control group
Rauchmann [30], Germany	DELCODE (multicentric, longitudinal, observational study)	Healthy elders and individuals at risk for AD (MF, 70.8 y)	938	Fish intake	EPIC-FFQ	Left and right hippocampal volume	MRI	Greater fish consumption was associated with increased bilateral hippocampal volumes (standardised regression coefficients: β = 0.09, *p* < 0.001 for left hippocampal volume and β = 0.09, *p* = 0.01 for right hippocampal volume)
Cui [41], UK	UK Biobank (prospective epidemiological study)	UK residents aged 40–69 y (MF, 52.7 y)	2723	Seafood intake (includes: oily fish, white fish and tinned tuna, breaded/battered fish)	24-hour recall	THV, RHV, LHV	MRI	Individuals with a higher intake of seafood exhibited higher longitudinal rates of change in THV, RHV, but not LHV compared to those with no consumption (THV, β: 0.004514; *p* < 0.05; RHV, β:0.005527, *p* < 0.05; LHV, β: 0.003269, *p* = 0.2929)
Feng [40]	UK Biobank (prospective epidemiological study), UK	UK individuals aged 40–69y (MF, 55.47 y)	30,375	Oily fish intake	Self-reported questionnaire data	WM BAG (white matter brain age gap)	DTI	Oily fish intake was associated with accelerated brain aging after adjustment for age, sex, and BMI (β = 0.08, BH-adjusted *p* < 0.001). A multivariate analysis including broad range of covariates confirmed this finding (β = 0.04, BH-adjusted *p* = 0.02)

Abbreviations: 3 C (Three-City); AD (Alzheimer’s disease); BAG (brain age gap); BDHQ (Brief Self-administered Diet History Questionnaire); CHS (Cardiovascular Health Study); CHS-CS (Cardiovascular Health Study Cognition Study); CT (Cortical Thickness); DELCODE (DZNE – Longitudinal Cognitive Impairment and Dementia Study); DTI (Diffusion Tensor Imaging); F (female); FA-BHQ (Fractional-anisotropy Brain Healthcare Quotient); FFQ (Food Frequency Questionnaire); GM-BHQ (Grey-matter Brain Healthcare Quotient); GMV (Grey Matter Volume); ICV (Intracranial Volume); IRR (incidence rate ratio); LHV (left hippocampus volume); M (male); MCI (mild cognitive impairment); MRI (Magnetic Resonance Imaging); MS (Multiple Sclerosis); MUCCA (Normalized mean upper cervical cord area); NBV (Normalized total brain volume); NCbV (Normalized cerebellar grey matter volume); NCGMV (Normalized white matter volume); NThalV (Normalized thalamic volume); NWMV (Normalized white matter volume); OR (odds ratio); RHV (Right hippocampus volume); SCD (subjective cognitive decline); SCG-FFQ (Scottish Collaborative Group Food Frequency Questionnaire); SQ-FFQ (Semiquantitative Food Frequency Questionnaire); TBV (total brain volume); TGMV (Total grey matter volume); THV (Total hippocampus volume); TWMV (Total white matter volume); WHIMS (Women’s Health Initiative Memory Study); WMG (White Matter Grade); WMH (White matter hyperintensities); WML (Lower total brain volume and greater white matter lesion volume), y (years)

In conclusion, this systematic review indicates that fish consumption is associated with several markers of preserved brain structure, with the strongest and most consistent findings emerging for white matter integrity, cerebrovascular health, and selective region-specific grey matter preservation in areas critical for memory and cognitive function. These associations are biologically plausible given the vasoprotective, anti-inflammatory, and neurotrophic properties of long-chain omega-3 fatty acids and other key nutrients found in fish. However, given the heterogeneity and methodological limitations identified across existing studies, future research should prioritize more rigorous, harmonized, and mechanistically informed approaches to clarify the relationship between fish consumption and brain structure. Longitudinal design, metabolic phenotyping (i.e., lipidomics, metabolomics) or biomarkers of fish intake (i.e., red blood cell DHA/EPA), implementation of genetic factors (i.e., FADS1/FADS2, APOE genotype), and focus on high-risk populations (i.e., older adults with hypertension, diabetes, or genetically elevated AD risk might strengthen the level of evidence and clarify whether fish intake confers differential neuroprotection under higher pathological burden. Notwithstanding, current findings reinforce the biological plausibility of fish-derived nutrients as modulators of vascular and myelin health and also emphasize the importance of focusing on small-vessel disease markers in future research. As white matter deterioration and microvascular injury are among the earliest and most consequential contributors to cognitive decline, understanding diet-based strategies that may attenuate these processes remains a key priority for brain aging research. These findings underscore the importance of considering fish consumption within the broader framework of whole-diet patterns, lifestyle behaviours, and individual biological differences. Overall, while fish consumption represents a promising, accessible dietary behaviour with potential to contribute to healthier brain aging, definitive conclusions require more harmonized, mechanistically guided, and methodologically robust research. Addressing these gaps will be essential for developing evidence-based dietary recommendations aimed at preserving brain structure and cognitive function across the lifespan.

## Supplementary Information

Below is the link to the electronic supplementary material.

Supplementary Material 1

## Data Availability

All data supporting the findings of this study are available from the corresponding author upon reasonable request.
