# Supplementary material for: Fish consumption and brain structure: a comprehensive systematic review of observational studies
Source: Aging Clin Exp Res. 2026 Apr 11;38(1):131. doi: 10.1007/s40520-026-03363-x (PMC13194236; doi:10.1007/s40520-026-03363-x)

Supplementary Table 1. The Meta-analysis of Observational Studies in Epidemiology (MOOSE) guidelines.

| **Section/topic** | **#** | **Checklist item** | **Reported on page #** |
| --- | --- | --- | --- |
| **TITLE** | | |  |
| Title | 1 | Identify the report as a systematic review, meta-analysis, or both. | 1 |
| **ABSTRACT** | | |  |
| Structured summary | 2 | Provide a structured summary including, as applicable: background; objectives; data sources; study eligibility criteria, participants, and interventions; study appraisal and synthesis methods; results; limitations; conclusions and implications of key findings; systematic review registration number. | 3 |
| **INTRODUCTION** | | |  |
| Rationale | 3 | Describe the rationale for the review in the context of what is already known. | 3,4 |
| Objectives | 4 | Provide an explicit statement of questions being addressed with reference to participants, interventions, comparisons, outcomes, and study design (PICOS). | 4 |
| **METHODS** | | |  |
| Protocol and registration | 5 | Indicate if a review protocol exists, if and where it can be accessed (e.g., Web address), and, if available, provide registration information including registration number. | 5 |
| Eligibility criteria | 6 | Specify study characteristics (e.g., PICOS, length of follow-up) and report characteristics (e.g., years considered, language, publication status) used as criteria for eligibility, giving rationale. | 5, Supplementary Table 3 |
| Information sources | 7 | Describe all information sources (e.g., databases with dates of coverage, contact with study authors to identify additional studies) in the search and date last searched. | 5 |
| Search | 8 | Present full electronic search strategy for at least one database, including any limits used, such that it could be repeated. | 5, Supplementary Table 2 |
| Study selection | 9 | State the process for selecting studies (i.e., screening, eligibility, included in systematic review, and, if applicable, included in the meta-analysis). | 5, Supplementary Figure 1 |
| Data collection process | 10 | Describe method of data extraction from reports (e.g., piloted forms, independently, in duplicate) and any processes for obtaining and confirming data from investigators. | 5 |
| Data items | 11 | List and define all variables for which data were sought (e.g., PICOS, funding sources) and any assumptions and simplifications made. | 5 |
| Risk of bias in individual studies | 12 | Describe methods used for assessing risk of bias of individual studies (including specification of whether this was done at the study or outcome level), and how this information is to be used in any data synthesis. | 5,6 |
| Summary measures | 13 | State the principal summary measures (e.g., risk ratio, difference in means). | NA |
| Synthesis of results | 14 | Describe the methods of handling data and combining results of studies, if done, including measures of consistency (e.g., I^2^) for each meta-analysis. | NA |
| Risk of bias across studies | 15 | Specify any assessment of risk of bias that may affect the cumulative evidence (e.g., publication bias, selective reporting within studies). | NA |
| Additional analyses | 16 | Describe methods of additional analyses (e.g., sensitivity or subgroup analyses, meta-regression), if done, indicating which were pre-specified. | NA |
| **RESULTS** | | |  |
| Study selection | 17 | Give numbers of studies screened, assessed for eligibility, and included in the review, with reasons for exclusions at each stage, ideally with a flow diagram. | 6, Supplementary Figure 1 |
| Study characteristics | 18 | For each study, present characteristics for which data were extracted (e.g., study size, PICOS, follow-up period) and provide the citations. | 7-12, Table 1 |
| Risk of bias within studies | 19 | Present data on risk of bias of each study and, if available, any outcome level assessment (see item 12). | Supplementary Table 4, Supplementary Table 5 |
| Results of individual studies | 20 | For all outcomes considered (benefits or harms), present, for each study: (a) simple summary data for each intervention group (b) effect estimates and confidence intervals, ideally with a forest plot. | NA |
| Synthesis of results | 21 | Present results of each meta-analysis done, including confidence intervals and measures of consistency. | NA |
| Risk of bias across studies | 22 | Present results of any assessment of risk of bias across studies (see Item 15). | NA |
| Additional analysis | 23 | Give results of additional analyses, if done (e.g., sensitivity or subgroup analyses, meta-regression [see Item 16]). | NA |
| **DISCUSSION** | | |  |
| Summary of evidence | 24 | Summarize the main findings including the strength of evidence for each main outcome; consider their relevance to key groups (e.g., healthcare providers, users, and policy makers). | 12-15 |
| Limitations | 25 | Discuss limitations at study and outcome level (e.g., risk of bias), and at review-level (e.g., incomplete retrieval of identified research, reporting bias). | 15, 16 |
| Conclusions | 26 | Provide a general interpretation of the results in the context of other evidence, and implications for future research. | 16,17 |
| **FUNDING** | | |  |
| Funding | 27 | Describe sources of funding for the systematic review and other support (e.g., supply of data); role of funders for the systematic review. | 17 |

Supplementary Table 2. Search strategy.

| Search strategy Pubmed | |
| --- | --- |
|  | (fish[title/abstract] OR seafood[title/abstract] OR shellfish[title/abstract]) AND ("neuroimaging"[title/abstract] OR MRI[title/abstract] OR "magnetic resonance imaging"[title/abstract] OR "brain volume"[title/abstract] OR "hippocampal volume"[title/abstract] OR "white matter"[title/abstract] OR "brain structure"[title/abstract] OR "gray matter"[title/abstract] OR microbleed*[title/abstract] OR "magnetic resonance imaging"[MeSH Terms]) |
| Search strategy Scopus | |
|  | ( TITLE-ABS ( fish ) OR TITLE-ABS ( seafood ) OR TITLE-ABS ( shellfish ) ) AND ( TITLE-ABS ( neuroimaging ) OR TITLE-ABS ( MRI ) OR TITLE-ABS ( "magnetic resonance imaging" ) OR TITLE-ABS ( "brain volume" ) OR TITLE-ABS ( "hippocampal volume" ) OR TITLE-ABS ( "white matter" ) OR TITLE-ABS ( "brain structure" ) OR TITLE-ABS ( "gray matter" ) OR TITLE-ABS ( microbleed* )) |

Supplementary Table 3. PICOS criteria.

| Parameter | Description |
| --- | --- |
| (P) Population | Adults |
| (I) Intervention/Exposure | Habitual fish, seafood or shellfish consumption |
| (C) Comparison | Different categories of habitual fish, seafood or shellfish consumption |
| (O) Outcome | Structural brain parameters |
| (S) Study design | Observational studies with a comparison group (cohort studies, cross-sectional studies, case-control studies) |

Supplementary Table 4. Assessment of study quality according to the Newcastle-Ottawa Quality Assessment Scale for cross-sectional studies

|  | SELECTION | | | COMPARABILITY | EXPOSURE | | TOTAL SCORE |
| --- | --- | --- | --- | --- | --- | --- | --- |
| Name, year | Representativeness of the exposed cohort | Non-respondents | Ascertainment of exposure |  | Assessment of outcome | Statistical test |  |
| Gardener, 2012 | ★ | - | ★ | ★★ | ★ | ★ | 6 |
| Raji, 2014 | ★ | - | ★ | ★★ | ★ | ★ | 6 |
| Gu, 2015 | ★ | - | ★ | ★★ | ★ | ★ | 6 |
| Del Brutto, 2016 | ★ | - | - | ★★ | ★ | ★ | 5 |
| Morris, 2016 | ★ | - | ★ | ★★ | ★ | ★ | 6 |
| Staubo, 2017 | ★ | ★ | ★ | ★★ | ★ | ★ | 7 |
| Croll, 2018 | ★ | - | ★ | ★★ | ★ | ★ | 6 |
| Kokubun, 2020 | - | - | ★ | ★★ | ★ | ★ | 5 |
| Ballarini, 2021 | ★ | - | ★ | ★★ | ★ | ★ | 6 |
| Del Brutto, 2021 | ★ | ★ | ★ | ★★ | ★ | ★ | 6 |
| Thomas, 2021 | ★ | ★ | - | ★★ | ★ | ★ | 6 |
| Loonstra, 2023 | ★ | - | - | ★★ | ★ | ★ | 5 |
| Rauchmann, 2023 | ★ | - | - | ★ | ★ | ★ | 4 |
| Feng, 2024 | ★ | - | - | ★★ | ★ | ★ | 5 |
|  |  |  |  |  |  |  |  |

Supplementary Table 5. Assessment of study quality according to the Newcastle-Ottawa Quality Assessment Scale for prospective studies

|  | SELECTION | | | | COMPARABILITY | EXPOSURE | | | TOTAL SCORE |
| --- | --- | --- | --- | --- | --- | --- | --- | --- | --- |
| Name, year | Representativeness of the exposed cohort | Selection of the non-exposed cohort | Ascertainment of exposure | Demonstration that outcome of interest was not present at start of study |  | Assessment of outcome | Was follow-up long enough for outcomes to occur | Adequacy of follow up of cohorts |  |
| Virtanen, 2008 | ★ | ★ | ★ | ★ | ★★ | ★ | ★ | ★ | 9 |
| Titova, 2013 | ★ | ★ | - | - | ★★ | ★ | ★ | ★ | 8 |
| Virtanen, 2013 | ★ | ★ | ★ | ★ | ★★ | ★ | ★ | ★ | 9 |
| Del C Valdés Hernández, 2017 | ★ | ★ | - | - | ★★ | ★ | ★ | ★ | 7 |
| Luciano, 2017 | ★ | ★ | - | ★ | ★★ | ★ | ★ | ★ | 8 |
| Chen, 2020 | ★ | ★ | ★ | - | ★★ | ★ | ★ | ★ | 8 |
| Klinedinst, 2021 | ★ | ★ | ★ | - | - | ★ | ★ | - | 5 |
| Del Brutto, 2022 | ★ | ★ | ★ | ★ | ★★ | ★ | ★ | - | 8 |
| Song, 2022 | ★ | ★ | ★ | ★ | ★★ | ★ | ★ | - | 8 |
| Cui, 2024 | ★ | ★ | - | ★ | ★★ | ★ | ★ | - | 7 |

Supplementary Figure 1.


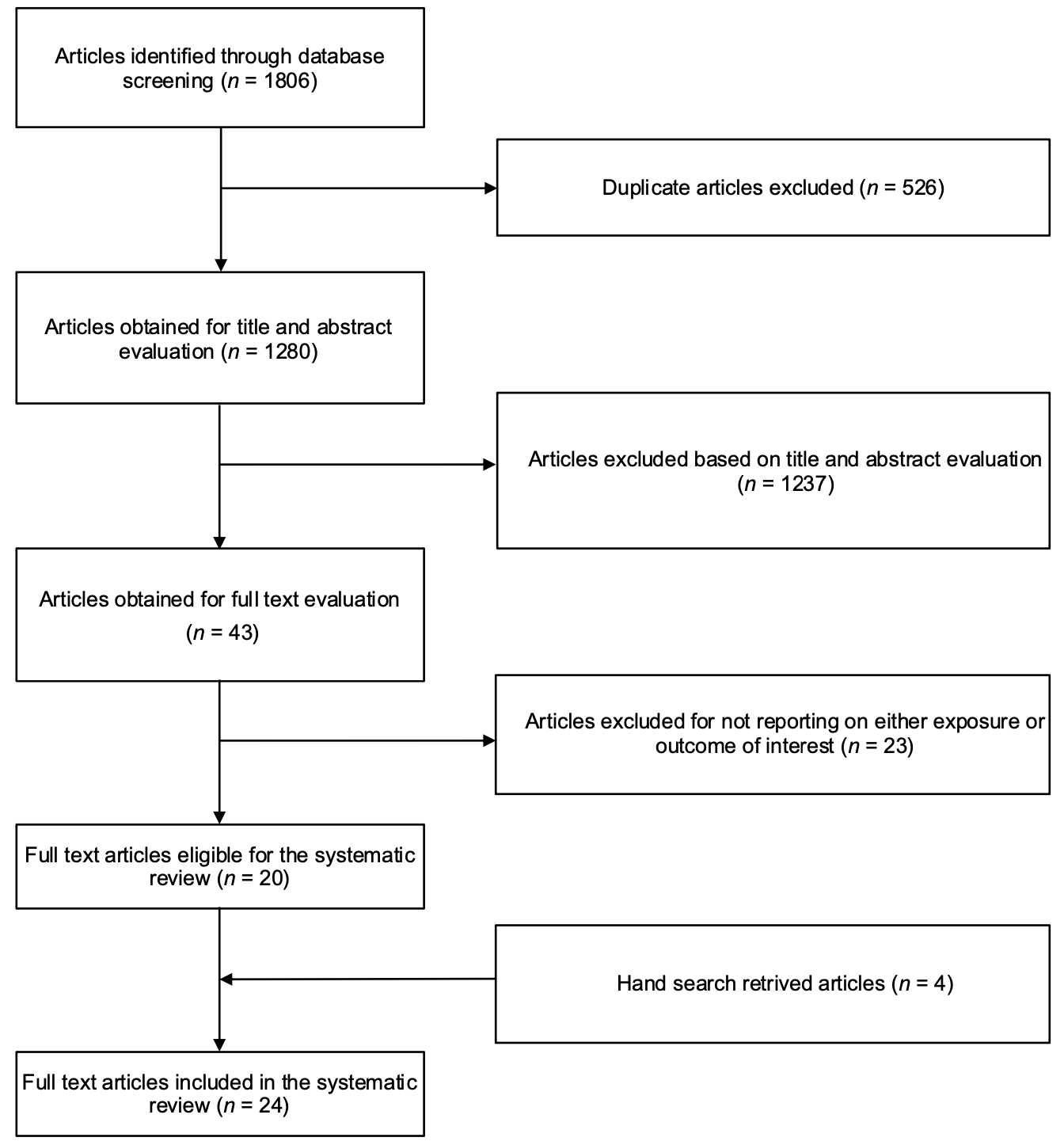

Supplement: Supplementary file 1 — Supplementary Material 1 [file 40520_2026_3363_MOESM1_ESM.docx]
